# Platinum-modified covalent triazine frameworks hybridized with carbon nanoparticles as methanol-tolerant oxygen reduction electrocatalysts

**DOI:** 10.1038/ncomms6040

**Published:** 2014-09-22

**Authors:** Kazuhide Kamiya, Ryo Kamai, Kazuhito Hashimoto, Shuji Nakanishi

**Affiliations:** 1 Department of Applied Chemistry, The University of Tokyo, 7-3-1 Hongo, Bunkyo-ku, Tokyo 113-8656, Japan; 2 Research Center for Advanced Science and Technology, The University of Tokyo, 1-5-3 Komaba, Meguro-ku, Tokyo 153-8904, Japan; 3 Core Technologies Development Center, Eco Solutions Company, Panasonic Corporation, 1048 Kadoma, Kadoma-city, Osaka 571-8686, Japan

## Abstract

Covalent triazine frameworks, which are crosslinked porous polymers with two-dimensional molecular structures, are promising materials for heterogeneous catalysts. However, the application of the frameworks as electrocatalysts has not been achieved to date because of their poor electrical conductivity. Here we report that platinum-modified covalent triazine frameworks hybridized with conductive carbon nanoparticles are successfully synthesized by introducing carbon nanoparticles during the polymerization process of covalent triazine frameworks. The resulting materials exhibit clear electrocatalytic activity for oxygen reduction reactions in acidic solutions. More interestingly, the platinum-modified covalent triazine frameworks show almost no activity for methanol oxidation, in contrast to commercial carbon-supported platinum. Thus, platinum-modified covalent triazine frameworks hybridized with carbon nanoparticles exhibit selective activity for oxygen reduction reactions even in the presence of high concentrations of methanol, which indicates potential utility as a cathode catalyst in direct methanol fuel cells.

## Abstract (web summary)

Covalent triazine frameworks are known catalysts for some catalytic reactions, but show no electrocatalytic activity. Here, the authors synthesize platinum modified covalent triazine frameworks hybridized with carbon nanoparticles, which are electro-active for oxygen reduction reactions.

## Graphical abstract

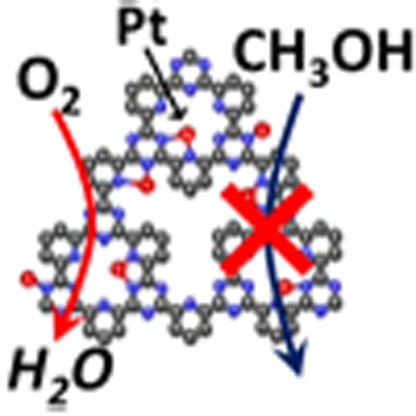

Covalent organic frameworks (COFs) have attracted a keen attention as novel catalyst platforms^1^,^2^,^3^,^4^,^5^,^6^ because of their unique physicochemical properties, including their nano-porous structure, mechanical robustness and high design flexibility. For example, Ding *et al.*^7^ reported that a palladium-modified imine-linked COF catalysed Suzuki–Miyaura coupling reactions with a high stability and an easy recyclability^7^. Chan-Thaw *et al.* deposited palladium nanoparticles on covalent triazine frameworks (CTFs) and demonstrated that this material was an effective catalyst for liquid-phase glycerol oxidation^8^. Another interesting example of a COF-based catalyst is platinum-modified CTF (Pt-CTF) developed by Palkovits *et al.*^9^,^10^, which has been known to function as a heterogeneous (solid) catalyst for partial oxidation of methane (CH_4_) to methylbisulfate (CH_3_OSO_3_H) in H_2_SO_4_ using oleum as an oxidant The Pt-CTF was developed by being inspired by Pt-bipyrimidine complex, known as a Periana catalyst, which could catalyse the same partial oxidation reaction^11^,^12^. Thus, the work carried out by Palkovits *et al.* is important in that they developed the concept of molecular complex catalysts to the robust heterogeneous Pt-CTF materials.

Pt-CTFs are attractive also as heterogeneous electrocatalysts, since molecular Pt complexes with a single metal centre is known to exhibit reaction selectivity in some homogeneous redox reactions, such as alkane oxidation^13^,^14^ and carbon dioxide reduction reactions^15^. Considering that molecular complex-based electrocatalysts are generally unstable, it is expected that Pt-CTFs can serve as electrocatalysts with both unique selectivity and high robustness. However, CTF-based materials have not previously been applied as electrocatalysts because of their poor electrical conductivity^16^. In the present work, we demonstrate a solution to this problem by synthesizing a hybrid material consisting of Pt-CTF and conductive carbon nanoparticles (CPs). The hybridized material (Pt-CTF/CP) exhibits electrocatalytic activity for oxygen reduction reaction (ORR) in acidic solution with a high methanol tolerance, which is a very attractive property for direct methanol fuel cells (DMFCs) as the methanol-crossover effect is one of the issues to be addressed^17^,^18^,^19^,^20^,^21^. To the best of our knowledge, this is the first demonstration of the potential for CTF-based materials to serve as electrocatalysts.

## Results

### Morphological characterization of the Pt-CTF/CP

**Figure 1:**
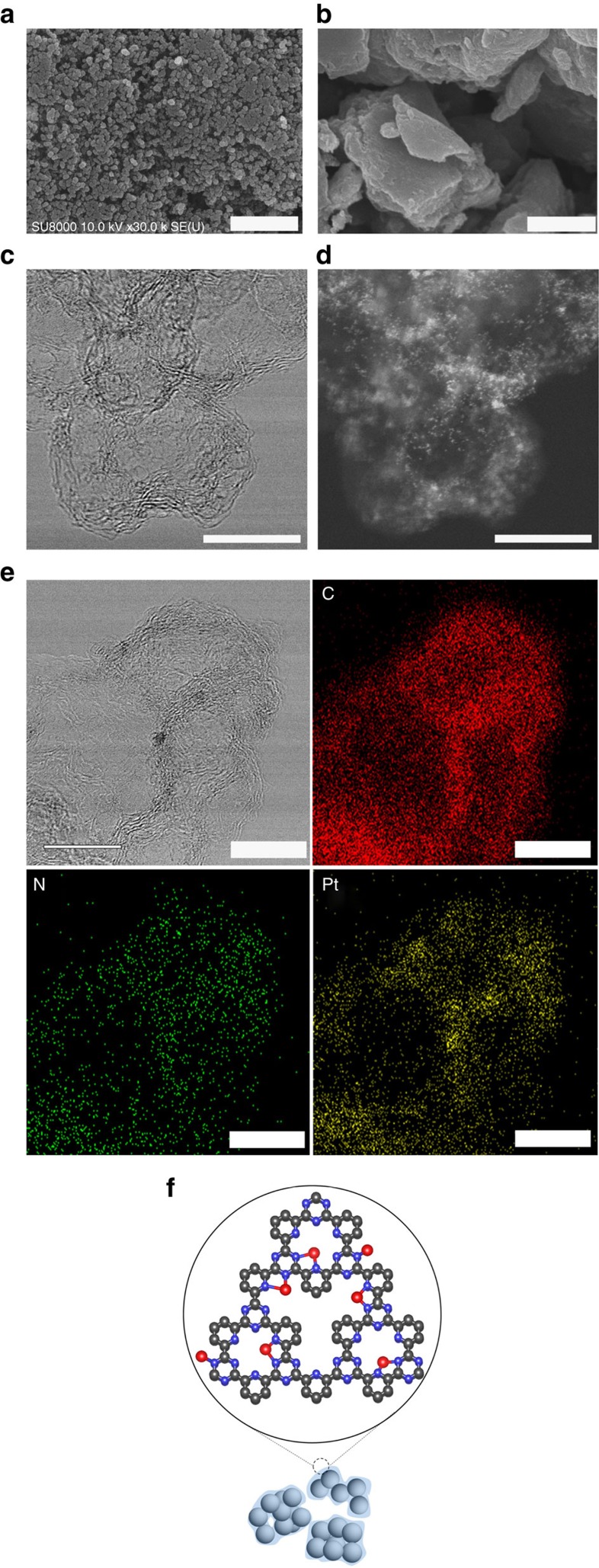
Electron microscopic images of Pt-CTF/CP. Representative SEM images of (**a**) Pt-CTF/CP and (**b**) Pt-CTF. (**c**) A representative high-resolution transmission electron microscopy (HR-TEM) image of Pt-CTF/CP and (**d**) the corresponding high-angle annular dark-field scanning transmission electron microscopy image. (**e**) Another HR-TEM image of Pt-CTF/CP and the corresponding EDX mappings for C, N and Pt atoms. Scale bar, 1 μm (**a**,**b**) and 10 nm (**c**–**e**). (**f**) Schematic illustration of Pt-CTF/CP (blue: N, red: Pt and black: C, Chlorine atoms are not shown for clarity).

Pt-CTF/CP was synthesized by modifying the standard synthesis protocol of Pt-CTF^9^,^10^,^22^,^23^. Briefly, CTFs were obtained by *in situ* polymerization of 2,6-dicyanopyridinein-molten ZnCl_2_ containing CPs (the weight ratio of 2,6-dicyanopyridine to CP was 1: 1), after which the CTF/CP was impregnated with a platinum chloride salt to obtain Pt-CTF/CP. Scanning electron microscopic (SEM) inspection reveals that the particles of Pt-CTF/CP (20–200 nm, Fig. 1a) are much smaller than that of Pt-CTF polymerized without CPs (1–5 μm, Fig. 1b), suggesting that CTF is well mixed with CPs during the *in situ* polymerization. Then, we conducted high-resolution transmission electron microscopy (Fig. 1c) and the corresponding high-angle annular dark-field scanning transmission electron microscopy (Fig. 1d), the latter of which is a powerful tool for discerning individual heavy atoms^24^,^25^,^26^. It was confirmed that the bright spots corresponding to Pt atoms (the sizes <0.5 nm) were uniformly dispersed and almost no Pt nanoparticles (the sizes >1 nm) were observed (Fig. 1d). Such bright spots could not be observed on the CPs (without Pt-CTF) as shown in Supplementary Fig. 1. Figure 1e exhibits another high-resolution transmission electron microscopy image and the corresponding elemental maps (carbon, nitrogen and platinum) obtained by energy dispersive X-ray (EDX) technique. Notably, the EDX maps revealed that Pt and N atoms are localized at the edges of CPs, strongly suggesting that CPs (or the aggregates) are covered with Pt-CTF as schematically shown in Fig. 1f.

Next, the nitrogen adsorption–desorption isotherms were obtained to analyse the pore structure of Pt-CTF/CP (Supplementary Fig. 2). Type IV isotherm and H_2_ hysteresis loop were observed, suggesting that porous structures existed in the synthesized Pt-CTF/CP^27^,^28^. The pore-size distribution calculated based on nonlocal density functional theory was shown in the inset of Supplementary Fig. 2. Although CP (Ketchenblack) is known to exhibit a peak at 3.6–3.7 nm (refs 29, 30), the synthesized Pt-CTF/CP exhibited peaks at 1.4 and 5.3 nm. The total pore volume and the BET surface area were estimated to be 0.79 cm^3 ^g^−1^ and 555 m^2 ^g^−1^, respectively.

### Electrochemical characterizations of Pt-CTF/CP

**Figure 2:**
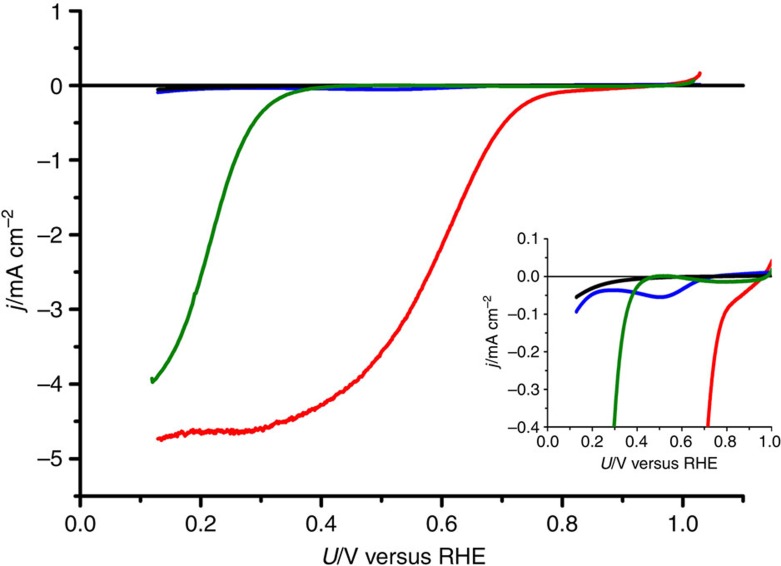
ORR electrocatalytic activities. *j* versus *U* curves for CTF (black), CTF/CP (green), Pt-CTF (blue) and Pt-CTF/CP (red) in 0.5 M H_2_SO_4_ saturated with dissolved oxygen, obtained at a scan rate of 10 mV s^−1^. Rotation rate 1,500 r.p.m. (Inset) Magnified curves.

Figure 2 shows current density (*j*) versus potential (*U*) curves for Pt-CTF/CP obtained in an oxygen-saturated 0.5 M H_2_SO_4_ solution. Although the ORR activity of Pt-CTF (without CP) was very low (blue curve), the ORR current increased significantly upon hybridization of Pt-CTF with CPs (red curve). This enhancement in ORR activity can be explained on the basis that both the electric conductivity and the electrochemically active surface area of the material were increased by the hybridization with CPs (Fig. 1a,b and Supplementary Table 1). In contrast, when the CTFs (without Pt) were hybridized with CPs (green curve), the ORR onset potential was much negative than that of Pt-CTF/CP, indicating that the Pt atoms in Pt-CTF/CP are an active centre for ORR. To the best of our knowledge, this is the first demonstration of the application of a CTF-based material as an electrocatalyst.

**Figure 3:**
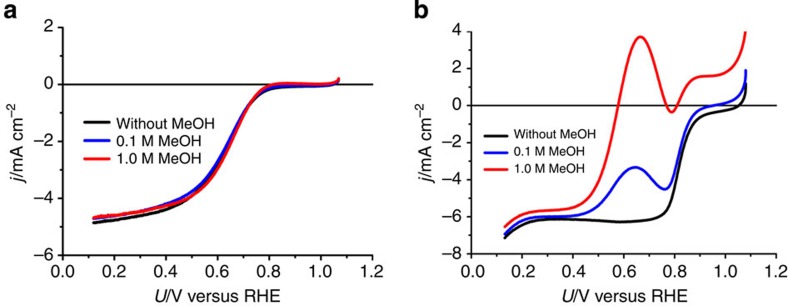
ORR activities of Pt-CTF/CP and Pt/C in the presence of methanol. *j* versus *U* curves for (**a**) Pt-CTF/CP and (**b**) 20 wt% Pt/C in 0.5 M H_2_SO_4_ saturated with dissolved oxygen. Methanol concentration: (black) 0 M, (blue) 0.1 M and (red) 1.0 M.

**Figure 4:**
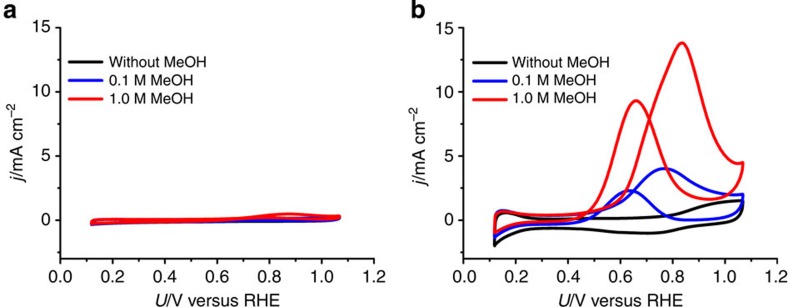
Methanol oxidation activity of Pt-CTF/CP and Pt/C. *j* versus *U* curves for (**a**) Pt-CTF/CP and (**b**) 20 wt% Pt/C in 0.5 M H_2_SO_4_ in the absence of oxygen. Methanol concentration: (black) 0 M, (blue) 0.1 M and (red) 1.0 M.

Next, to investigate the methanol tolerance of Pt-CTF/CP, we intentionally added methanol to a 0.5-M H_2_SO_4_ solution in the presence of oxygen. Cyclic voltammograms obtained in the presence of methanol are shown in Fig. 3. In case of a commercial 20 wt% Pt/C, the oxidation peak of methanol can be clearly observed at around +600 mV versus RHE (Fig. 3b). After the addition of 1 M methanol, the onset potential of the cathodic current shifted ~200 mV in the negative direction, reaching 580 mV versus RHE. In contrast, surprisingly, the overlap of the methanol oxidation current with that of the ORR was almost negligible for Pt-CTF/CP even in the presence of 1 M methanol. To directly compare the methanol oxidation activity of Pt-CTF/CP and 20 wt% Pt/C, we obtained cyclic voltammograms in H_2_SO_4_ solution containing methanol in the absence of dissolved oxygen (Fig. 4). The peak currents for methanol oxidation with Pt-CTF/CP (Fig. 4a) were ~1/40 compared with those with Pt/C (Fig. 4b). We confirmed that methanol oxidation is inactive even in 0.5 M HClO_4_ as shown in Supplementary Fig. 3, which excludes the possibility that the methanol tolerance originated from the suppression of methanol oxidation by strongly adsorbed sulfate/bisulfate^31^,^32^. Thus, the above results clearly showed that Pt-CTF/CP exhibits little activity with regard to methanol oxidation.

### X-ray characterizations of Pt-CTF/CP

Characterizations of Pt-CTF/CP were conducted using various X-ray techniques to obtain information on the molecular mechanism of the methanol tolerance. The surface concentration of each element was determined by an X-ray photoelectron spectroscopy (XPS), and the results are summarized in Table 1. Peaks assignable to Pt and N were confirmed, and the Pt/N elemental ratio did not show a clear dependence on the amount of CP (the right column of Table 1), implying that the structures of Pt-CTFs are essentially identical even by hybridizing with CPs. In addition, the surface concentration of C, which was calculated with XPS, became higher with the increasing ratio of CP. Taking into account that the CP aggregates are uniformly covered by Pt-CTFs (Fig. 1), these results indicated that the thickness of Pt-CTFs is less than the escape depth of photoelectrons (ca. ~3 nm) as schematically shown in Fig. 1f, which enabled the Pt-CTF to possess electronic conduction with the CPs.

The details of the electronic structures of Pt-CTFs were investigated by taking XPS and X-ray absorption near-edge structures (XANES; Supplementary Figs 4–7). The Pt-4f_7/2_ peaks were located at 72.5 eV for Pt-CTF/CP and 72.2 eV for Pt-CTF (Supplementary Fig. 4). These binding energies were over 1 eV higher than those for metal Pt (70.9 eV), revealing that the valence state of Pt was Pt(II)^33^,^34^. In addition, XANES of Pt L_3_ edge shown in Supplementary Fig. 5 exhibited that the white-line intensities at 11,562 eV for Pt-CTF (1.45) and Pt-CTF/CP (1.46) are higher than those for metal Pt (1.24), indicating that Pt atoms existed as oxidized forms in both Pt-CTF and Pt-CTF/CP^35^. Thus, the Pt-4f XPS and the XANES results also show that there was no formation of Pt aggregates (metal Pt) on Pt-CTFs. Next, we focused on the N-1s XPS spectrum (Supplementary Fig. 6 and Supplementary Table 2). The N-1s peak at 399.2 eV observed for the 2,6-dicyanopyridine (that is, the catalyst monomer, Supplementary Fig. 7) was not observed for the Pt-CTF/CP, indicating that the cyclical trimerization of cyano reaction groups efficiently proceeded. The N-1s peak of Pt-CTF/CP could be deconvoluted into C_2_NH (398–399 eV) and C_3_N (400–401 eV)^4^. The peak assigned to C_2_NH was shifted to the higher-energy side upon Pt loading on both CTF and CTF/CP, indicating that the electron density of the N atoms became lower in the presence of Pt atoms. The decrease in the electron density of the N atoms can be explained by considering the formation of Pt-N coordination bonds (this point will be argued later). All the features described above were confirmed even for Pt-CTF (that is, without CPs), indicating that the hybridization with CPs did not influence on the electronic properties of N atoms in Pt-CTFs.

**Figure 5:**
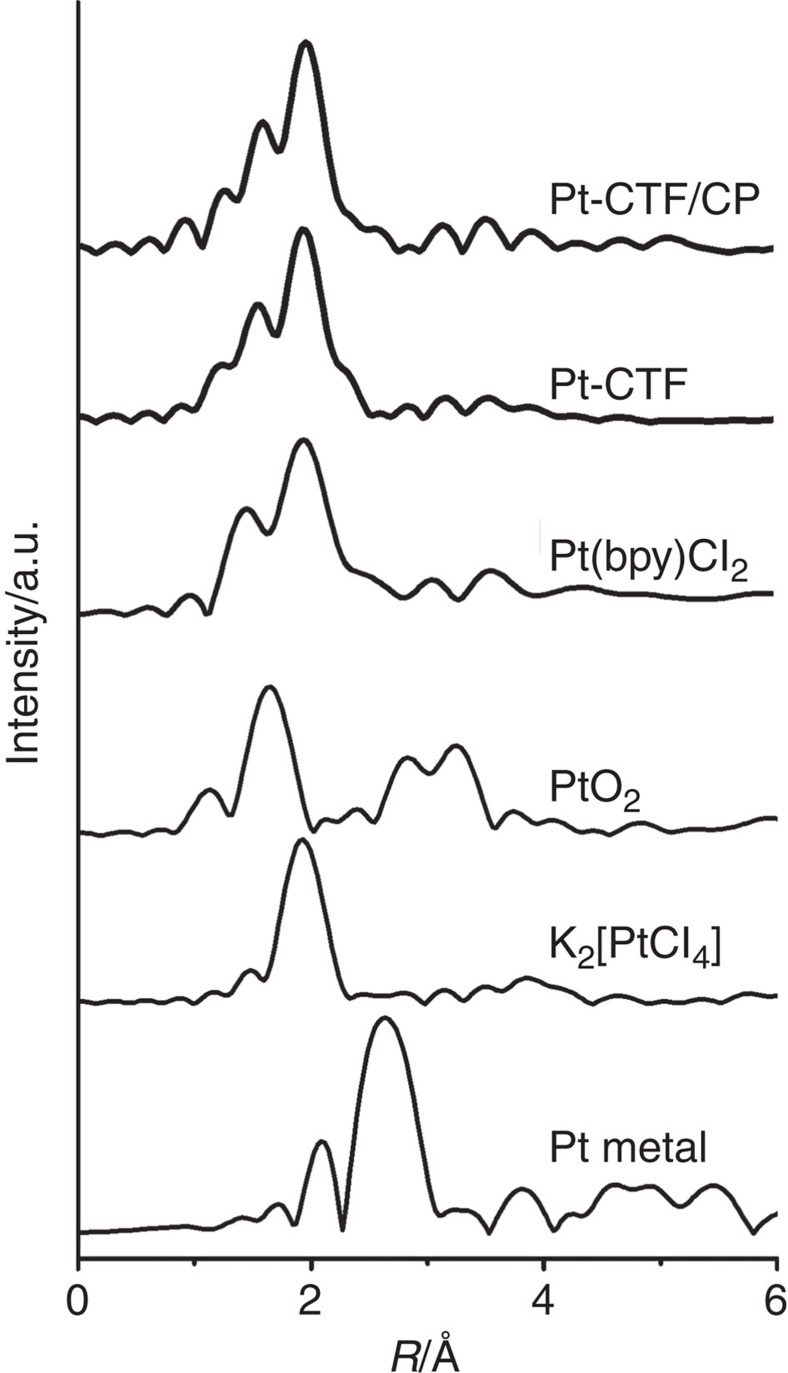
EXAFS analyses. *k*^*3*^-weighted Fourier transform spectra of EXAFS of Pt L_3_ edge for Pt-CTF/CP and Pt-CTF. The spectra of Pt(bpy)Cl_2_, PtO_2_, K_2_[PtCl_4_] and Pt metal are also shown as reference.

Next, we conducted extended X-ray absorption fine structure (EXAFS) analyses of Pt L_3_ edge to obtain information on the molecular structure of Pt-CTF/CP. Fourier transformations of *k*_3_-weighted EXAFS oscillations for Pt-CTF/CPs, Pt-CTFs, commercial Pt(bpy)Cl_2_ (bpy: 2,2'-bipyridine), PtO_2_, K_2_PtCl_4_ and Pt metal are shown in Fig. 5. The peak corresponding to a Pt–Pt bond at 2.6 Å was not observed at all for Pt-CTF/CPs. Instead, two peaks at *R=*1.5 and 1.9 Å assignable to Pt–N and Pt–Cl bonds, respectively, were clearly observed, indicating that Pt exists in the form of a single atom as illustrated in Fig. 1f. The ratio of the Pt–N peak to the Pt–Cl peak for Pt-CTFs corresponded to that for Pt(bpy)Cl_2_ (model PtN_2_Cl_2_ complex), implying that Pt atoms mainly existed as PtN_2_Cl_2_. It should be noted here that there was no clear difference in the EXAFS spectra between Pt-CTF/CP and Pt-CTF, indicating that the molecular structure of Pt-CTF is maintained upon the hybridization with CPs.

## Discussion

Let us consider here about the molecular mechanism of the methanol-tolerant ORR electrocatalytic activity of Pt-CTF/CP. First, we consider about the ORR on a single Pt site. An oxygen molecule is known to adsorb on a single Pt atom through either the Griffiths model (side-on adsorption) or the Pauling model (end-on adsorption)^36^. After the adsorption, *π* donation from the oxygen 2*π* orbitals to the unoccupied Pt 5*d* orbitals, and the *π* back donation from occupied Pt 5*d* orbitals to the oxygen 2π* orbitals could simultaneously occur, resulting in the O–O bond activation^36^,^37^. In fact, Nakamura *et al.*^38^ revealed using ^18^O isotopic infrared study that a single Pt atom and an oxygen molecule exchanged electrons, forming Pt–O_2_ adduct through the side-on adsorption. Li *et al.*^39^ also demonstrated by using first-principles calculations that the O–O bond was activated on single Pt site via the *π* back donation through the end-on adsorption, resulting in the weakening of the O–O bond. These reported lines of work suggested that electrocatalytic ORR could proceed even on a single Pt atom.

On the contrary, a number of researchers have suggested that at least two Pt atoms are needed to oxidize methanol^40^,^41^,^42^,^43^,^44^,^45^,^46^. It is well known that methanol can be oxidized via a dual-path mechanism on Pt, that is, the carbon monoxide (CO) pathway and the non-CO pathway^44^,^45^,^47^. Cuesta^45^,^48^ revealed by using cyanide-modified Pt electrode that dehydrogenation reaction of methanol to CO, a critical step of the CO pathway, required at least three contiguous Pt sites. As for the non-CO pathway, Osawa and colleagues^44^ proposed a molecular mechanism based on the results obtained from *in situ* infrared (IR) absorption spectroscopy. They suggested that adsorbed oxygenated species (that is, O_ad_ or OH_ad_) on the Pt site next to the methanol absorption site are needed for first dehydrogenation of the O–H bond in methanol. This molecular mechanism was further supported by Kuzume *et al.*^49^ again by *in situ* IR absorption spectroscopy. The previous lines of work have thus revealed that at least two adjacent Pt sites are required for methanol oxidation regardless of whether the reaction proceeds via the CO or the non-CO pathways. Actually, to the best of our knowledge, there have been no reports of methanol oxidation on a single Pt-atom site. Considering these literatures, the methanol tolerance of Pt/CTF is reasonably explained because the EXAFS results demonstrated that the Pt atoms in Pt/CTF were isolated.

In conclusion, we successfully synthesized Pt-CTF hybridized with CP, preserving its electronic and molecular structure, which exhibited ORR activity in acidic solutions with a high methanol tolerance. The methanol tolerance of the Pt-CTF/CP indicates that the methanol-crossover effect can be ignored. This property allows a considerable increase in the concentration of methanol in DMFC; consequently, the energy density of DMFC is expected to increase because of the use of Pt-CTF/CP. Another interesting aspect of this work is that it is the first demonstration of electrocatalytic function in CTF-based materials. This was achieved by hybridizing non-conductive CTFs with conductive CPs. We anticipate that this methodology could be applied generally to other CTFs with unique catalytic properties.

## Methods

### Synthesis procedure of Pt-CTF/CP

CTF/CP was synthesized by polymerization of 2,6-dicyanopyridine in a molten salt of zinc chloride (ZnCl_2_). Specifically, 1.363 g ZnCl_2_ (Wako), 0.129 g 2,6-dicyanopyridine (Sigma) and 0.129 g KetchenBlack EC600JD were mixed in a globe box. The mixture was placed in a Pyrex glass tube and the tube was sealed, evacuated, heated and kept at a terminal temperature of 400 °C for 21 h. The resulting powder was washed with 0.1 M HCl, water, tetrahydrofolate and acetonitrile, and dried *in vacuo*. Pt atoms were modified by impregnation in 160 mM K_2_[PtCl_4_] (Wako) aqueous solution at 60 °C for 4 h before being washed with water and acetone.

### Electrochemical characterization

Electrochemical activity for ORR was evaluated using a rotating disk electrode. Working electrodes were first prepared by dispersing 5 mg of powder samples in 175 μl ethanol and 47.5 μl Nafion solution (5 wt% solution in a mixture of lower aliphatic alcohols and water, Aldrich). A 7-μl aliquot of ink was dropped on a glassy carbon electrode (0.196 cm^−2^). The catalyst loading was controlled at 0.8 mg cm^−2^. A Pt wire and Ag/AgCl (saturated KCl) were used as the counter and reference electrodes, respectively. Commercial 20 wt% Pt/C and Pt-Vulcan XC-72 were purchased from Tanaka Kikinzoku and Fuel Cell Earth, respectively. The electrical conductivity of the catalysts was measured with a resistivity meter (Mitsubishi Chemical, Loresta GP). The working electrode was rotated with a rotation speed of 1,500 r.p.m.

### Physical characterizations

X-ray photoelectron spectra (Axis Ultra, Kratos Analytical Co.) were taken with monochromated Al Kα X-rays at *hν*=1,486.6 eV. For detailed chemical analysis, backgrounds of core-level spectra were subtracted using the Shirley method. N-1s spectra were fitted with Voigt (70% Gaussian and 30% Lorentzian) functions. Hard X-ray absorption measurements (XAFS) were performed at the hard X-ray beam line BL01B01 at SPring-8, Japan and by using R-XAS Looper (Rigaku). Transmission-yield spectra were acquired using a double-crystal Si (111) monochromator. The morphological structures were characterized using TEM with an EDX detector (ARM-200F, JEOL) and SEM (SU-8000, Hitachi). The nitrogen adsorption–desorption isotherm at 77 K was obtained by a BET (NOVA-4200e, Quantachrome).

## Author contributions

K.K., R.K. and S.N. designed the experiments and performed the analyses. K.K., K.H. and S.N. wrote the manuscript.

## Additional information

**How to cite this article:** Kamiya, K. *et al.* Platinum-modified covalent triazine frameworks hybridized with carbon nanoparticles as methanol-tolerant oxygen reduction electrocatalysts. *Nat. Commun.* 5:5040 doi: 10.1038/ncomms6040 (2014).

## Supplementary Material

Supplementary Information Supplementary Figures 1-7 and Supplementary Tables 1-2.

## Figures and Tables

**Table 1:** XPS-elemental analyses.

	**C**	**N**	**Pt**	**Cl**	**Pt/N**
CTF	81	17	—	2.6	—
Pt-CTF	76	15	3.0	5.5	0.20
Pt-CTF/CP	91	5.6	1.0	2.3	0.18

CP, carbon nanoparticle; CTF, covalent triazine framework; Pt-CTF, platinum-modified CTF; XPS, X-ray photoelectron spectroscopy.

The composition ratios (atomic %) for CTF, Pt-CTF and Pt-CTF/CP estimated from the XPS results.
