# Supplementary material for: Platinum-modified covalent triazine frameworks hybridized with carbon nanoparticles as methanol-tolerant oxygen reduction electrocatalysts
Source: Nat Commun. 2014 Sep 22;5:5040. doi: 10.1038/ncomms6040 (PMC4199112; doi:10.1038/ncomms6040)
Supplement: Supplementary Information — Supplementary Figures 1-7 and Supplementary Tables 1-2. [file ncomms6040-s1.pdf]

Supplementary Information

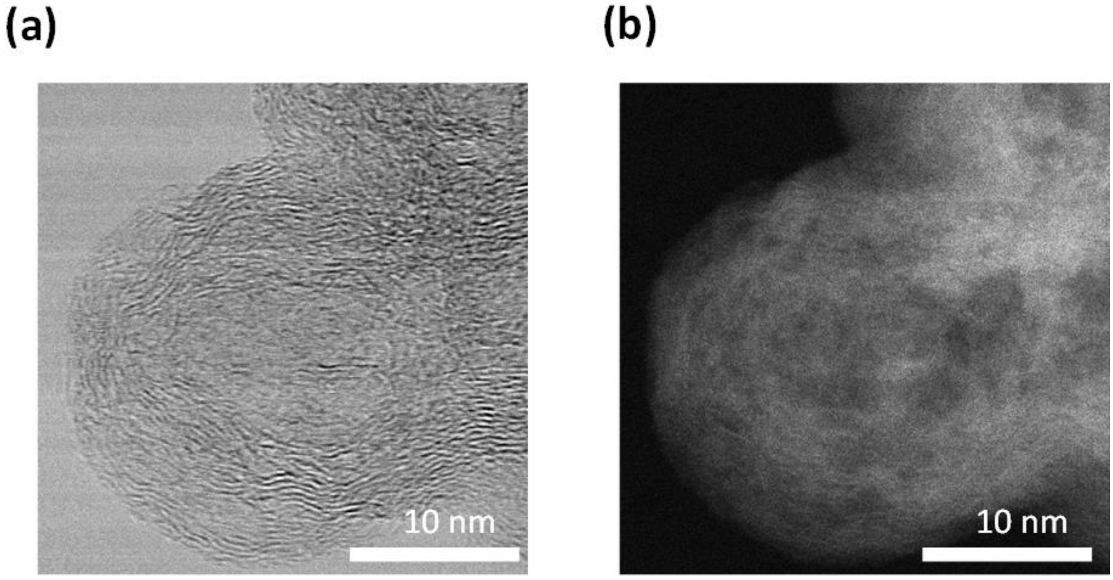

Supplementary Fig. 1 (a) High-resolution and (b) high-angle annular dark-field scanning transmission electron microscopic images of CP.

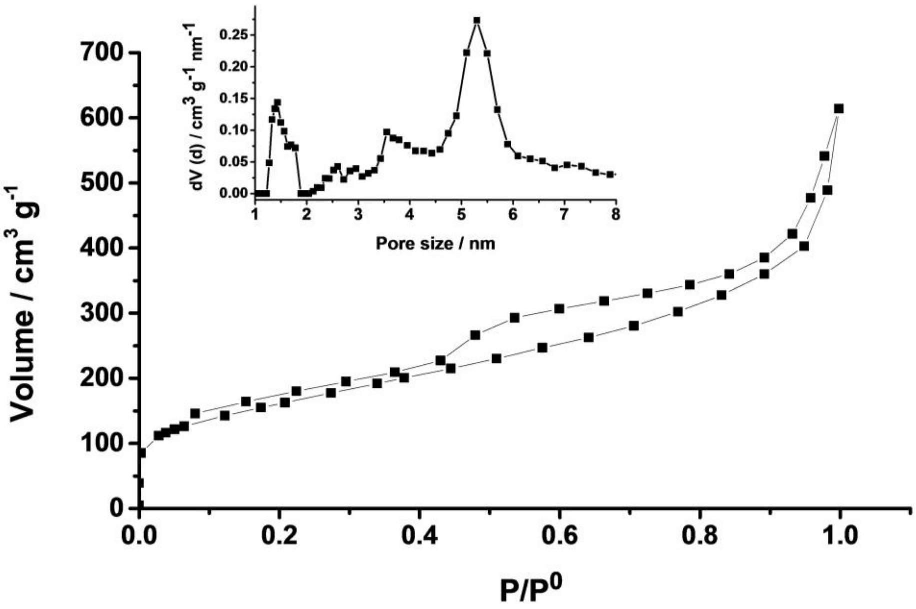

Supplementary Fig. 2 Nitrogen adsorption-desorption isotherms for Pt-CTF/CP. The inset shows the pore size distribution calculated based on nonlocal density functional theory (NL-DFT).

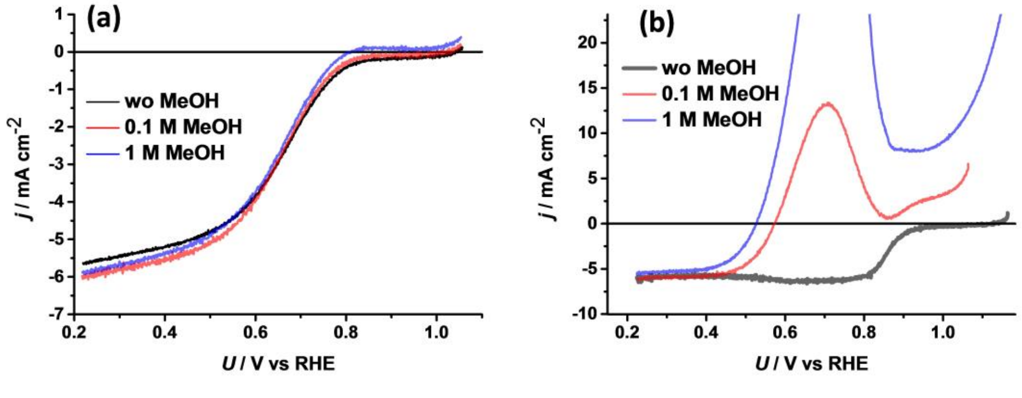

Supplementary Fig. 3 *j* vs. *U* curves for (a) Pt-CTF/CP and (b) 20 wt % Pt-Vulcan XC-72 in 0.5 M $HClO_4$ saturated with dissolved oxygen. Methanol concentration: (black) 0 M, (red) 0.1 M, and (blue) 1.0 M. The scan rates were 5 mV / s.

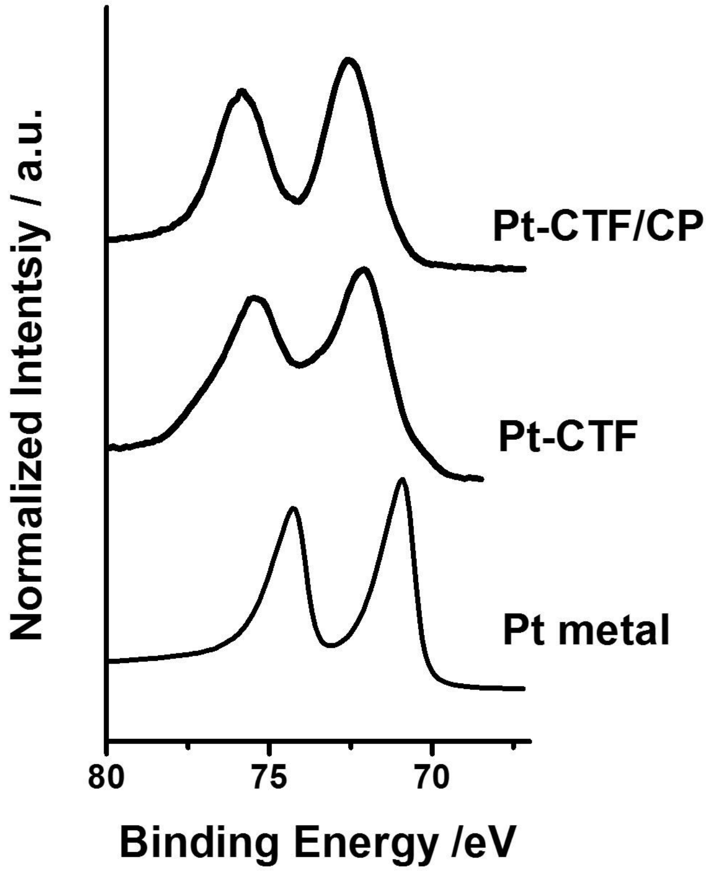

Supplementary Fig. 4 Pt-4f XPS spectra for Pt-CTF/CP, Pt-CTF, and Pt metal.

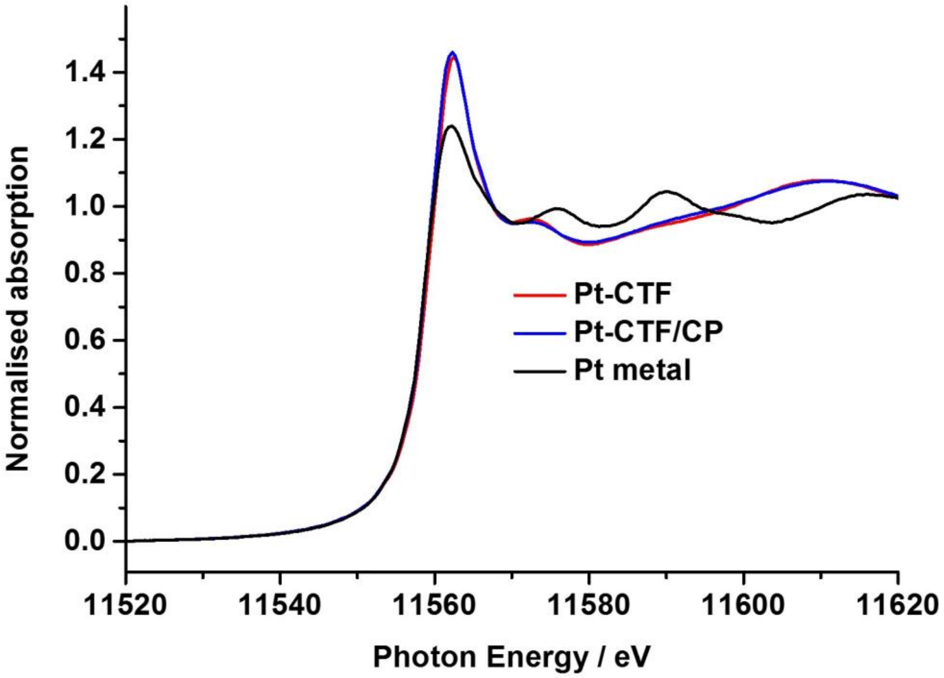

Supplementary Fig. 5 Pt L_3 XANES spectra for Pt-CTF, Pt-CTF/CP, and Pt metal.

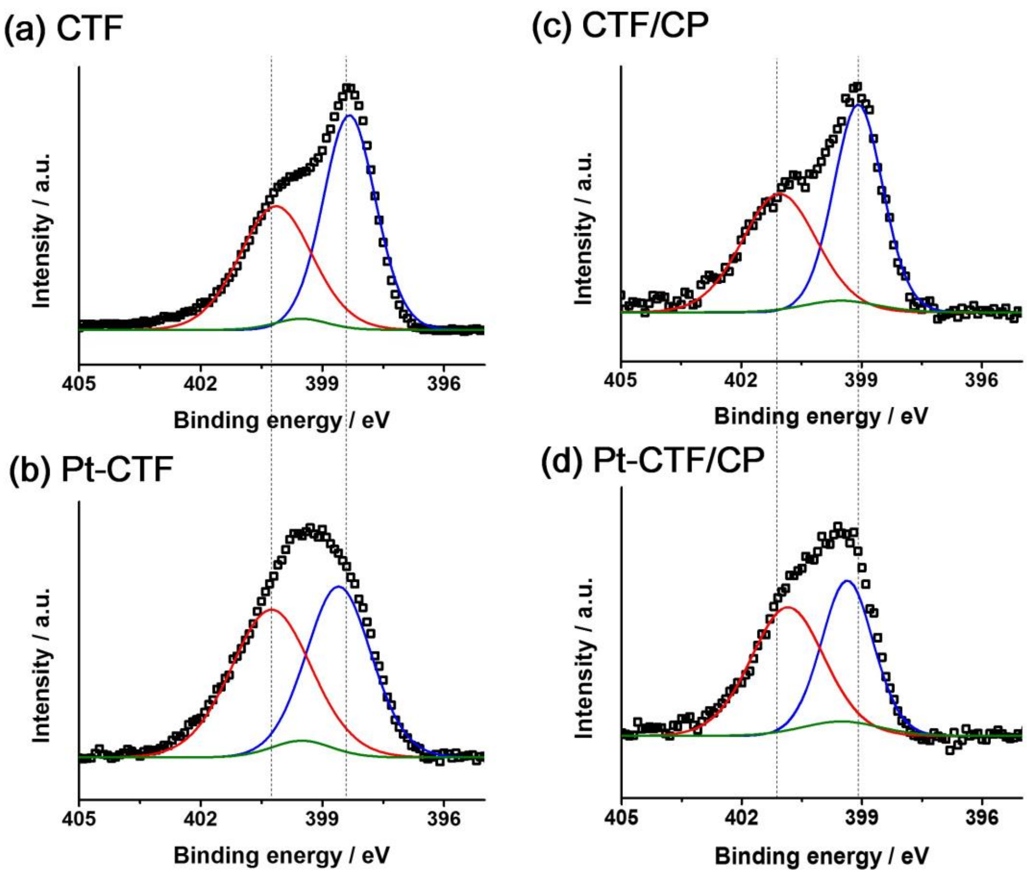

Supplementary Fig. 6 N-1s XPS spectra for (a) CTF, (b) Pt-CTF, (c) CTF/CP and (d) Pt-CTF/CP, Black dots, measured data; lines, deconvoluted curves (blue; C_2NH, red; C_3N, green; cyano group). Note that the peak positions were not identical upon adding CP as CTF is not conductive and easily to be charged by X-ray irradiation.

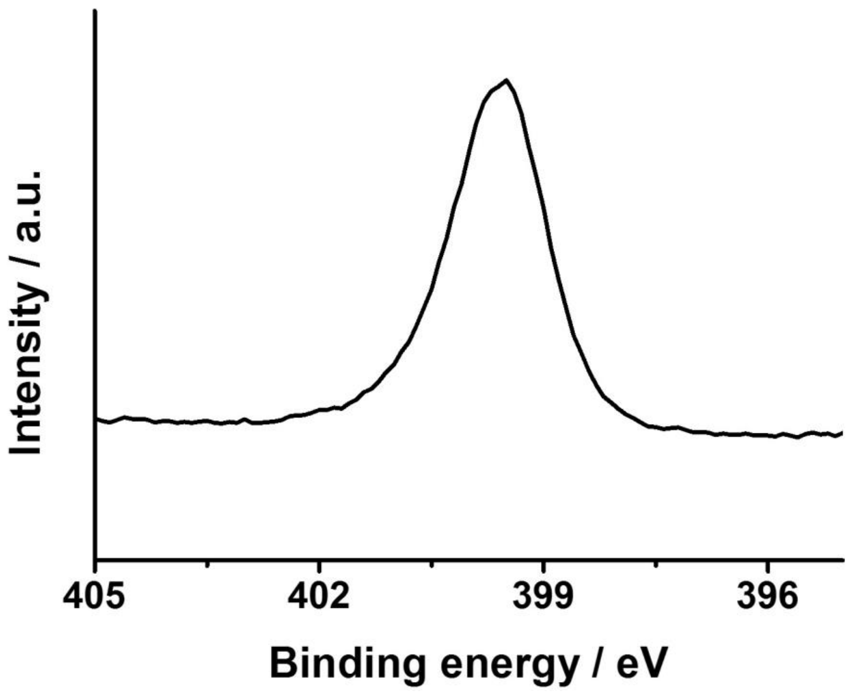

Supplementary Fig. 7 N-1s XPS spectra for 2,6-dicyanopyridine.

	Resistance [Ω cm]
Pt-CTF	> 10^6
Pt-CTF/CP	14
20 wt% Pt/C	3.8

Supplementary Table 1 Electrical conductivities of Pt-CTF, Pt-CTF/CP and Pt/C.

**(a) CTF**	Binding energy / eV	Relative ratio / %
C_2NH	398.4	61.5
C_3N	400.1	35.3
C≡N	399.5	3.2

**(b) Pt-CTF**	Binding energy / eV	Relative ratio / %
C_2NH	398.6	51.0
C_3N	400.2	44.0
C≡N	399.5	5.0

**(c) CTF/CP**	Binding energy / eV	Relative ratio / %
C_2NH	399.1	61.6
C_3N	401.0	35.0
C≡N	399.5	3.5

**(d) Pt-CTF/CP**	Binding energy / eV	Relative ratio / %
C_2NH	399.4	52.1
C_3N	400.9	43.1
C≡N	399.5	4.8

Supplementary Table 2 Binding energies and relative ratios of the four nitrogen components derived from decomposed N 1s XPS spectra.
